# Serum small extracellular vesicle‐derived *LINC00853* as a novel diagnostic marker for early hepatocellular carcinoma

**DOI:** 10.1002/1878-0261.12745

**Published:** 2020-07-13

**Authors:** Soon Sun Kim, Geum Ok Baek, Hye Ri Ahn, Suna Sung, Chul Won Seo, Hyo Jung Cho, Suk Woo Nam, Jae Youn Cheong, Jung Woo Eun

**Affiliations:** ^1^ Department of Gastroenterology Ajou University School of Medicine Suwon South Korea; ^2^ Department of Biomedical Sciences Ajou University Graduate School of Medicine Suwon South Korea; ^3^ Functional RNomics Research Center The Catholic University of Korea Seoul South Korea; ^4^ Department of Biomedicine & Health Sciences Graduate School of Medicine The Catholic University of Korea Seoul South Korea

**Keywords:** biomarker, extracellular vesicles, hepatocellular carcinoma, *LINC00853*, long noncoding RNA

## Abstract

This study aimed to identify novel long noncoding RNA (lncRNA) biomarkers for hepatocellular carcinoma (HCC) using publicly available tissue genomic datasets and validate their diagnostic utility for early‐stage HCC. Differentially expressed lncRNAs between 371 HCC and 50 nontumor tissues were obtained from The Cancer Genome Atlas liver hepatocellular carcinoma (TCGA_LIHC) project. Subsequently, the expression of the serum‐ and extracellular vesicle (EV)‐derived lncRNA was assessed in 10 patients with HCC and 10 healthy controls using RT–qPCR. The candidate lncRNAs were validated in 90 HCC and 92 non‐HCC (29 healthy control, 28 chronic hepatitis, 35 liver cirrhosis) patients. The sensitivity, specificity, and area under the receiver operating characteristic curve (AUC) were calculated for the candidate lncRNAs and the current HCC biomarker, alpha‐fetoprotein (AFP). *SFTA1P, HOTTIP, HAGLROS, LINC01419, HAGLR, CRNDE*, and *LINC00853* were markedly upregulated in HCC in TCGA_LIHC dataset. Among them, *LINC00853* has not been reported in relation to HCC before. In patients with HCC, only expression of small EV‐derived *LINC00853* (EV‐*LINC00853)* was increased. EV‐*LINC00853* showed excellent discriminatory ability in the diagnosis of all‐stage HCC (AUC = 0.934, 95% confidence interval = 0.887–0.966). Moreover, using a 14‐fold increase and 20 ng·mL^−1^ as cutoffs for EV‐*LINC00853* expression and AFP level, respectively, EV‐*LINC00853* was found to have a sensitivity of 93.75% and specificity of 89.77%, while AFP showed only 9.38% sensitivity and 72.73% specificity for the diagnosis of early‐stage HCC (mUICC stage I). EV‐*LINC00853* had a positivity of 97% and 67% in AFP‐negative and AFP‐positive early HCC, respectively. Serum EV‐derived *LINC00853* may be a novel potential diagnostic biomarker for early HCC, especially for AFP‐negative HCC.

## Graphical abstract

Long noncoding RNA (lncRNA) dysregulation has been observed in several cancers, including liver cancer. Here, we show that extracellular vesicle (EV)‐derived lncRNA EV‐*LINC00853* showed high sensitivity and specificity for diagnosis of early HCC. EV‐*LINC00853* is a potential diagnostic biomarker for early HCC, especially for AFP‐negative HCC.

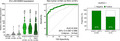

**Abbreviations**
AFP: alpha‐fetoprotein
ANOVA: one‐way analysis of variance
AUC: area under the curve
CH: chronic hepatitis
CI: confidence interval
EDS: extracellular vesicle‐depleted serum
EV: extracellular vesicle
HCC: hepatocellular carcinoma
LC: liver cirrhosis
lncRNA: long noncoding RNA
mUICC: modified Union for International Cancer Control
NTA: nanoparticle tracking analysis
ROC: receiver operating characteristic
RT–qPCR: quantitative reverse transcription PCR
SD: standard deviation
TCGA_LIHC: The Cancer Genome Atlas liver hepatocellular carcinoma
TEM: transmission electron microscopy

## 1 Introduction

Liver cancer is the sixth most prevalent cancer and fourth most common cause of cancer‐related death globally; the high mortality rate is mainly due to the late diagnosis and poor response to therapy [1]. Hepatocellular carcinoma (HCC) accounts for ~ 90% of the primary liver cancers and represents a major global health problem. Approximately 90% of the HCCs are associated with a known underlying etiology, most frequently chronic viral hepatitis B or C, excessive alcohol intake, or aflatoxin exposure [2]. Individuals at high risk of developing HCC are recommended to undergo abdominal ultrasonography every 6 months [3]. However, ultrasonography has only a sensitivity of 63% for detecting early‐stage HCC [4]. Alpha‐fetoprotein (AFP), which is the most widely used blood biomarker in HCC, also shows suboptimal performance as a serological test in HCC surveillance because of fluctuations in the AFP levels during hepatitis flares and 10–20% positivity in early‐stage HCC [5]. Therefore, there is an urgent need to develop better screening tools and diagnostic tests for diagnosis of early‐stage HCC to improve the prognosis of patients with this fatal disease.

Long noncoding RNAs (lncRNAs) are referred transcripts having a lengths exceeding 200 nucleotides that do not encode proteins in general; they are related to various functions, including regulation of transcription in *cis* or *trans*, modulation of messenger RNA (mRNA) processing, post‐translational control of protein activity, and organization of nuclear domains [6, 7]. Many lncRNAs have been functionally associated with human diseases [8], and their dysregulation has been observed in several cancers, including liver cancer [9, 10, 11]. Altered lncRNA expression can contribute to cancer phenotypes by stimulating cellular proliferation, angiogenesis, immune evasion, metastasis, and inhibiting apoptosis [8, 12].

Small extracellular vesicles (EVs; < 100 nm) play key roles in numerous normal and pathological biological processes [13]. Small EVs transfer proteins, DNA, and various forms of RNA, such as microRNA (miRNA), lncRNA, and mRNA, between tumor and nontumor cells [14]. Various EV‐derived lncRNAs, including lncRNA‐*HEIH, LINC02394, LINC0635, LINC00161,* and *JPX* [15, 16, 17, 18], were recently reported as diagnostic biomarkers for HCC. However, research into the diagnostic potential of lncRNAs in HCC has been limited by small sample sizes and unsatisfactory diagnostic performance for early‐stage HCC.

The present study aimed to identify novel HCC‐related lncRNAs using publicly available tissue genomic datasets and validate their diagnostic performance for early‐stage HCC in a moderately large cohort of patients with different liver diseases.

## 2 Materials and methods

### 2.1 Resources of publicly available genomic data

To investigate the expression of lncRNA biomarkers in HCC, genomic data were acquired from The Cancer Genome Atlas liver HCC project (TCGA_LIHC, https://cancergenome.nih.gov) and the GEO database of the NCBI (Accession Numbers: GSE94660, GSE114584, and GSE124535). The expression data for each lncRNA were log^2^ transformed [log^2^(FPKM + 1)] for downstream analyses.

### 2.2 Gene set enrichment analysis

To investigate gene signatures that were enriched from known molecular databases, we downloaded gene sets from MSigDB (http://software.broadinstitute.org/gsea/msigdb) at the Broad Institute Gene Set Enrichment Analysis (http://www.broadinstitute.org/gsea).

### 2.3 Patient enrollment and clinical term definitions

Patients were enrolled from the Ajou University Hospital, Suwon, South Korea, between January 2014 and December 2018. The study subjects were allocated into one of four groups: healthy control, chronic hepatitis (CH), liver cirrhosis (LC), and HCC. A healthy control was defined as an individual between 18 and 50 years of age without any medical history, who visited the Ajou Health Promotion Center for health check‐up. CH was diagnosed based on the persistence of serum hepatitis B surface antigen or hepatitis C virus RNA for more than 6 months. LC was diagnosed based on ultrasonographic findings including splenomegaly, blunt angle, and morphological changes [19]. HCC was diagnosed if tumor had a maximum diameter > 1 cm and characteristic features of HCC (arterial phase hyperenhancement, washout in the portal venous or delayed phase, threshold growth, and capsule appearance) in multiphase computed tomography and/or magnetic resonance imaging. If these criteria were present but there was a lack of diagnostic certainty, then a liver biopsy was performed to confirm the diagnosis of HCC [20]. Early‐stage HCC was defined as a single lesion less than 2 cm in diameter corresponding to the modified Union for International Cancer Control (mUICC) stage I. The test cohort consisted of 10 patients with HCC and 10 healthy controls, and the validation cohort consisted of 90 patients with HCC and 92 patients without HCC (29 healthy controls, 28 with CH, and 35 with LC). Patients whose AFP level measurements were unavailable were excluded from the comparative analysis. Overall survival was defined as the time from HCC diagnosis to death resulting from any causes. All investigations performed in the present study were conducted in accordance with the guidelines of the 1975 Declaration of Helsinki. The study protocol was approved by the Institutional Review Board of the Ajou University Hospital, Suwon, South Korea (AJRIB‐BMR‐KSP‐18‐397 and AJIRB‐BMR‐KSP‐18‐299). Anonymous serum samples and clinical data were provided by the Ajou Human Bio‐Resource Bank. Informed consent was waived.

### 2.4 Cell culture

Huh7 cells (Korean Cell Line Bank, Seoul, Korea) were cultured in Dulbecco's modified Eagle's medium (GenDEPOT, Barker, TX, USA) supplemented with 10% FBS (Invitrogen, Waltham, MA, USA) and 100 U·mL^−1^ penicillin–streptomycin (GenDEPOT), at 37° C in a humidified incubator with 5% CO_2_.

### 2.5 Separation of blood serum

Five milliliters of blood was collected from each individual directly into serum collection tubes. The blood was centrifuged at 1800 ***g*** for 10 min to extract the serum, which was aliquoted into 1.5‐mL tubes and stored at −80 °C. The serum samples were centrifuged at 3000 ***g*** at 4 °C for 15 min to remove cell debris before analysis.

### 2.6 Characterization of serum small EVs

Small EVs were extracted from the serum using ExoQuick (System Biosciences, Mountain View, CA, USA) according to the manufacturer's instructions with minor modifications [21]. Briefly, serum samples (300 μL) were mixed with ExoQuick (72 μL) and incubated at 4 °C overnight. The mixtures were then centrifuged at 1500 ***g*** for 30 min at room temperature. The supernatants were collected and used as EV‐depleted serum (EDS), whereas the pellets were resuspended in PBS (100 μL) and stored at −80 °C for subsequent extraction of RNA and proteins.

Transmission electron microscopy (TEM), nanoparticle tracking analysis (NTA), and western blotting were performed to confirm the presence and size of small EVs. For TEM, small EVs were marked with 10‐nm gold particles conjugated to anti‐CD63 antibody. Sample fixation was performed with 2% glutaraldehyde and 4% paraformaldehyde for 2 h at room temperature, and the EVs were inspected under a Sigma 500 electron microscope (Carl Zeiss, Jena, Germany). The size and quantity of the isolated EVs were examined using the NanoSight NS300 instrument (Malvern Panalytical Ltd., Malvern, UK) equipped with a 405 nm laser. A 60‐s video was recorded with a frame rate of 30 frames/s, and the particle movement was evaluated using nta software (version 3.0, Malvern Panalytical). Each sample was analyzed three times, and the counts were merged.

For western blotting, EDS, serum derived‐small EVs, and Huh7 total cell lysate were lysed in RIPA lysis buffer (100 μL; Thermo Scientific, Waltham, MA, USA) and incubated on ice for 10 min. Total protein concentration was quantified by the bicinchoninic acid assay (Thermo Scientific). The proteins (10 μg) were separated on 4–20% Mini‐PROTEAN TGX™ gels (Bio‐Rad, Hercules, CA, USA) and then transferred to poly(vinylidene difluoride) membranes (Amersham; GE Healthcare, Munich, Germany). The membranes were blocked in 5% nonfat milk in TBS‐T and immunoblotted using the following primary antibodies: mouse anti‐CD81 (1 : 250; 10630D; Invitrogen), rabbit anti‐CD9 (1 : 2000; ab92726; Abcam, Cambridge, UK), mouse anti‐ALIX (1 : 1000; sc‐53538; Santa Cruz Biotechnology, Dallas, TX, USA), mouse anti‐HSP90 (1 : 1000; SMC‐149; StressMarq Biosciences Inc., Victoria, BC, Canada), mouse anti‐BiP/GRP78 (1 : 1000; 610979; BD Biosciences, San Jose, CA, USA), and rabbit anti‐APOA1 (1 : 1000; ab52945; Abcam). The samples were then probed with secondary HRP‐conjugated anti‐rabbit (BR170‐6515; Bio‐Rad) or anti‐mouse (BR170‐6516; Bio‐Rad) antibodies.

### 2.7 Isolation of serum RNA and small EV‐derived RNA from peripheral blood samples from patients

RNA from serum‐derived EVs was extracted using the SeraMir™ Exosome RNA Amplification Kit (System Biosciences) according to the manufacturer's instructions. Briefly, serum samples (300 μL) were mixed with ExoQuick solution (72 μL) and incubated at 4 °C overnight. The mixtures were centrifuged at 1500 ***g*** for 30 min at room temperature before the supernatants were removed and the pellets resuspended in PBS (100 μL). The EV lysates were mixed with lysis buffer (300 μL) and 100% EtOH (200 μL). After vortexing for 10 s, the mixtures were transferred to a spin column and centrifuged at 15 928 ***g*** for 1 min and then washed twice with wash buffer (400 μL). After further centrifugation for 2 min, small EV‐derived RNA was eluted in elution buffer (30 μL).

Serum RNA was isolated using the TRIzol‐LS reagent (Invitrogen). In brief, serum (300 μL) was lysed in TRIzol‐LS (900 μL) before the RNA was phase separated using chloroform (240 μL), precipitated with 100% isopropanol, and washed in 75% EtOH. Finally, the RNA was eluted in RNase‐free water (30 μL).

The RNA concentration was assessed using the NanoDrop 2000 spectrophotometer (Thermo Scientific), while its yield and size distribution were analyzed using the Agilent 2100 Bioanalyzer and RNA 6000 Nano kit (Agilent Technologies, Foster City, CA, USA).

### 2.8 Quantitative reverse transcription PCR (RT–qPCR)

The expression level of the serum‐derived and the serum small EV‐derived lncRNA was measured using RT–qPCR. Serum RNA (500 ng) was reverse transcribed into complimentary DNA (cDNA) using the PrimeScript™ RT Master mix (TaKaRa Bio, Otsu, Japan), whereas small EV‐derived RNA (500 ng) was reverse transcribed using the miScript II RT kit (QIAGEN, Hilden, Germany). The resultant cDNAs were used as templates for RT–qPCR with the amfiSure qGreen Q‐PCR Master Mix (GenDEPOT), which was monitored in real time using the ABI 7300 Real‐Time PCR System (Applied Biosystems™, Foster City, CA, USA). PCR conditions were as follows: 15 s at 95 °C for denaturation, 34 s at 60 °C for primer annealing, and 30 s at 72 °C for primer extension. The following primer pairs were used as follows: LINC00853 forward: AAAGGCTAGGCGATCCCACA, reverse: ACTCCCTAGCTTGGCTCTCCT; HMBS forward: GGAGGGCAGAAGGAAGAAAACAG, reverse: CACTGTCCGTCTGTATGCGAG. The $2^{‐\Delta \Delta C_{t}}$ method was used to determine target gene expression relative to the internal control gene, *HMBS*. Relative *LINC00853* levels were calculated using $2^{‐\Delta \Delta C_{t}}$, where ΔC*_t_* = C*_t_* (*LINC00853*) − C*_t_* (*HMBS*) and ΔΔC*_t_* = ΔC*_t_* (individual samples) − ΔC*_t_* (mean of normal samples). All measurements were performed in triplicate.

### 2.9 Statistical analysis

The data are presented as mean ± SD of three experiments. All statistical analyses were performed in IBM spss version 22.0 (SPSS Inc., Chicago, IL, USA) and graphpad prism version 7.01 (GraphPad Software, San Diego, CA, USA). For numerical variables, one‐way analysis of variance (ANOVA) with Tukey's *post hoc* analysis was used to perform multiple comparisons between the three groups. The associations between categorical parameters were assessed using the two‐sided chi‐square test. Survival curves were plotted using the Kaplan–Meier method, and significant differences between the curves were determined using log‐rank test. *P* values < 0.05 were considered to be statistically significant. Each candidate biomarker accuracy for HCC was assessed by the area under the curve (AUC), sensitivity, and specificity based on receiver operating characteristic (ROC) curves analysis. The Youden index was used to determine optimal cutoff values.

## 3 Results

### 3.1 Selection of candidate HCC‐associated lncRNAs

**Fig. 1:**
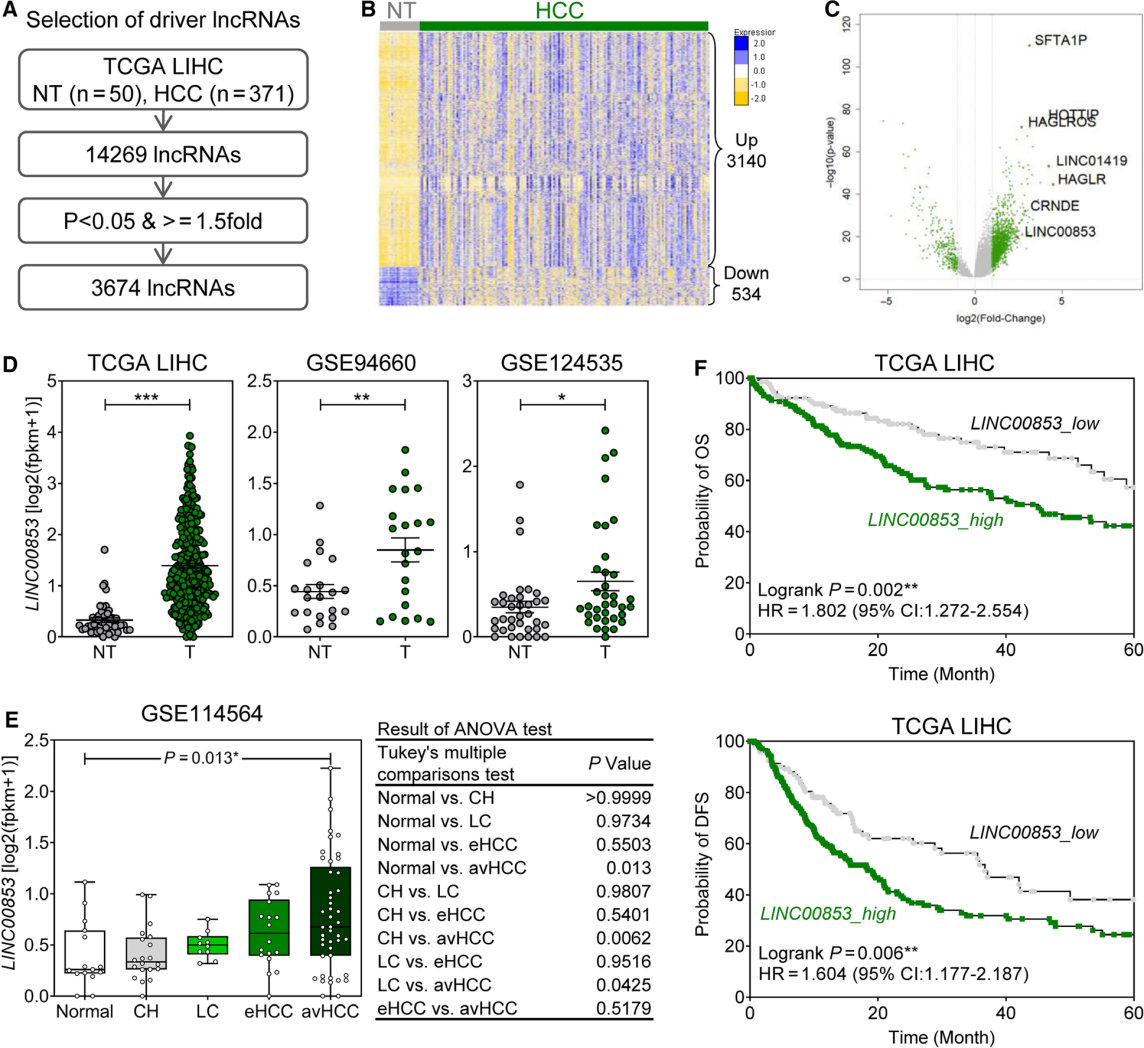
Correlation between clinical findings and *LINC00853* overexpression in HCC cohorts. (A) The strategy to identify novel lncRNA markers for HCC. (B) Heatmap of 3674 HCC‐associated lncRNA signatures in the TCGA_LIHC dataset. (C) Volcano plot representation of differentially expressed lncRNA signatures in the nontumor and the HCC cohorts. (D) *LINC00853* expression in the nontumor and the HCC cohorts in three HCC RNA‐Seq datasets (TCGA_LIHC, GSE94660, and GSE124535). Welch's *t*‐test; *P < 0.05, **P < 0.01, ***P < 0.001. (E) Changes in expression of 10 candidate marker genes in patients with different types and severity of liver disease in GSE114564 dataset. Statistically significant differences were determined using one‐way ANOVA with Tukey's multiple comparisons test. (F) The Kaplan–Meier survival curves for overall survival (left) and disease‐free survival (right) based on *LINC00853* expression in patients with HCC in TCGA_LIHC dataset.

In order to identify novel lncRNAs that play key roles in the development of HCC, we analyzed the publicly available lncRNA profiles of 371 HCC and 50 surrounding nontumor tissues from TCGA‐LIHC dataset (Fig. 1A). Among the 14 269 lncRNAs, 3674 were significantly differentially expressed between the HCC and nontumor specimens (*P* < 0.05 and ≥ 1.5‐fold change). Specifically, 3140 lncRNAs were upregulated while 534 lncRNAs were downregulated in HCC (Fig. 1B). Volcano plot analysis identified seven distinctly upregulated lncRNAs (*SFTA1P*,* HOTTIP*,* HAGLROS*,* LINC01419*,* HAGLR*,* CRNDE*, and *LINC00853*; Fig. 1C). According to a review of the literature (Table S1), we propose that *LINC00853* as a novel HCC‐related lncRNA that has not been reported thus far. We verified the expression of *LINC00853* in publicly available HCC RNA‐Seq datasets (TCGA_LIHC, GSE94660, GSE124535, and GSE114564) and found that it was not only consistently overexpressed in HCC in all three datasets, but also that its expression increased with the progression of liver disease to HCC (Fig. 1D, 1E, *P* = 0.0017). The remaining six lncRNAs were also significantly overexpressed in HCC (Fig. S1). Survival analysis based on *LINC00853* expression in TCGA_LIHC dataset showed that high *LINC00853* expression was associated with poor overall survival and disease‐free survival (Fig. 1F, log‐rank *P* = 0.002, *P* = 0.006, respectively).

### 3.2 Expression of *LINC00853* in the serum and serum small EVs in the test cohort

**Fig. 2:**
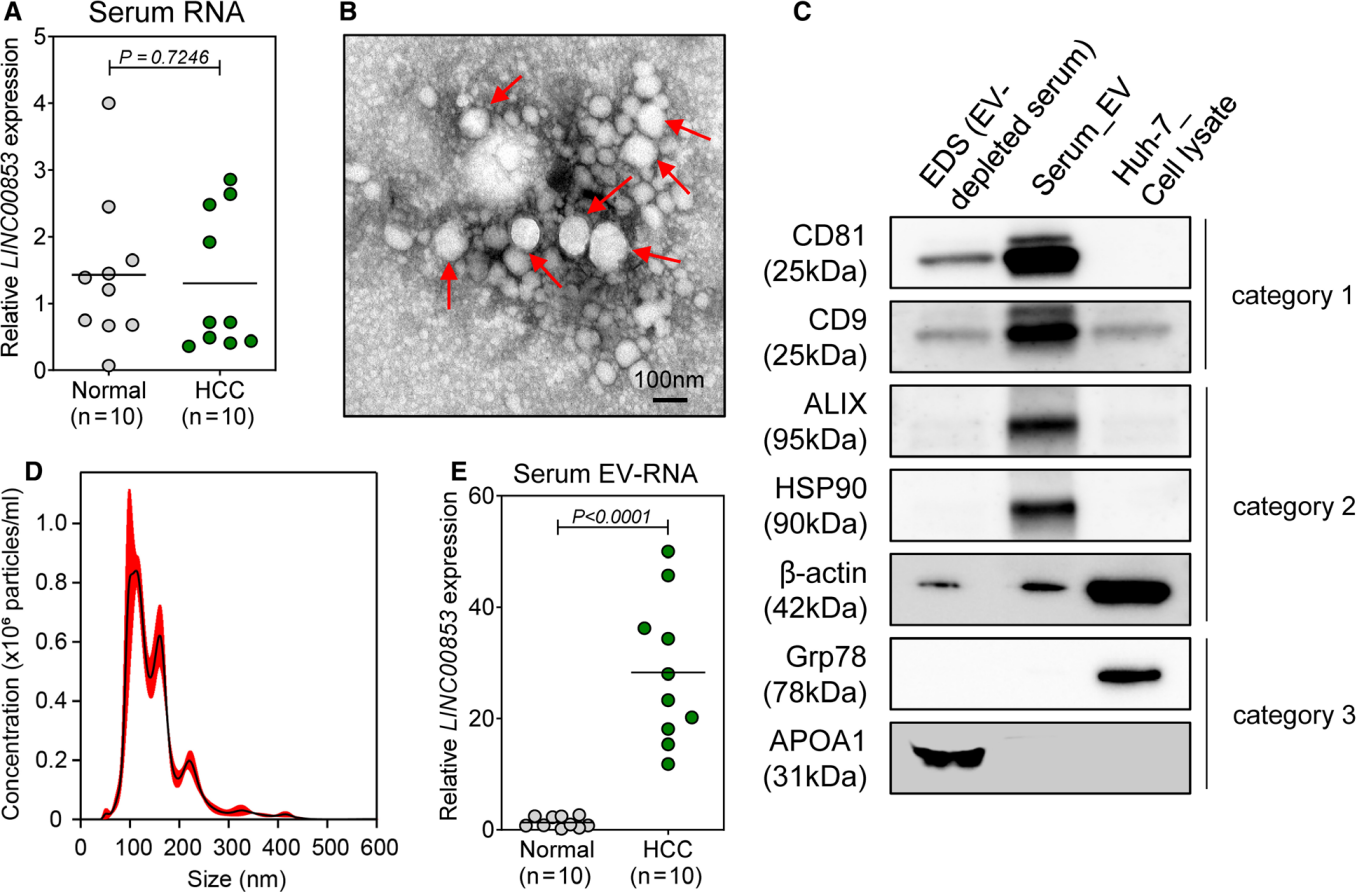
Expression of *LINC00853* in patient serum samples and serum‐derived EVs. (A) Scatter plot of *LINC00853* expression in the sera of healthy subjects (*n* = 10) and patients with HCC (*n* = 10). Statistically significant differences were determined using Welch's *t*‐test. Black horizontal lines denote means. (B) TEM image showing the spherical morphology of the isolated small EVs (diameter = ~ 100 nm), bar = 100 nm. (C) Representative immunoblots showing the expression of EV markers in the isolated EVs, according to MISEV 2018 guidelines. EDS and Huh‐7 cell lysate were used as controls. (D) The concentration and size distribution of EVs in the serum of a patient with HCC, as determined by NTA. (E) Scatter plot of *LINC00853* expression in serum‐derived EVs of healthy subjects (*n* = 10) and patients with HCC (*n* = 10). Statistically significant differences were determined using Welch's *t*‐test. Black horizontal lines denote sample means. Target gene expression was calculated relative to that of *HMBS*.

To evaluate the utility of *LINC00853* as a noninvasive diagnostic marker for HCC, we measured *LINC00853* expression in the serum (Fig. 2A) and serum EVs of 10 healthy controls and 10 patients with HCC. After separation from the serum, the EVs were characterized using TEM (Fig. 2B), immunoblotting for positive and negative protein markers of EV (Fig. 2C), and NTA (Fig. 2D). RT–qPCR analysis revealed that the level of serum‐derived *LINC00853* was similar in the two groups (Fig. 2A, *P* = 0.7246), whereas that of the serum EV‐derived *LINC00853* (EV‐*LINC00853)* was significantly higher in patients with HCC than in healthy controls (Fig. 2E,* P* < 0.001).

### 3.3 Validation of EV‐*LINC00853* as a diagnostic biomarker for HCC

**Fig. 3:**
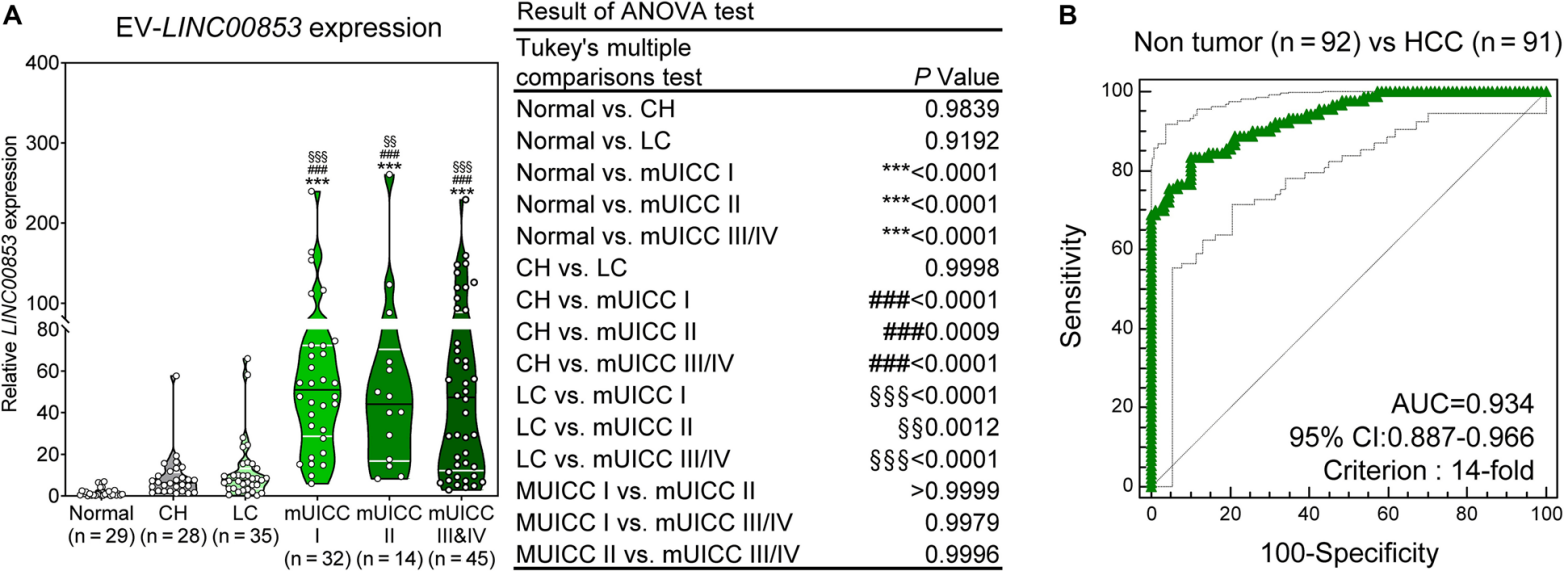
Expression of EV‐*LINC00853* and its diagnostic performance in the validation cohort. (A) Violin plot of EV‐*LINC00853* expression, as measured by RT–qPCR. Statistically significant differences were determined using the one‐way ANOVA with Tukey's multiple comparisons test. Black horizontal lines denote means, and error bars represent SEM. Compared to healthy liver; *P < 0.05, **P < 0.01, ***P < 0.001, compared to CH; #*P* < 0.05, ##*P* < 0.01, ###*P* < 0.001, compared to LC; §*P* < 0.05, §§*P* < 0.01, §§§*P* < 0.001. (B) Analysis of EV‐*LINC00853* ROC curve in patients with HCC vs control (healthy, CH, and LC). Statistically significant differences in the AUC were relative to AUC of 0.5. Target gene expression was calculated relative to that of *HMBS*.

A total of 90 patients with HCC and 89 patients without HCC were enrolled to validate the diagnostic performance of EV‐*LINC00853* for HCC. Demographic and clinical parameters of all subjects are listed in Table 1. The most common etiology of CH, LC, and HCC was hepatitis B virus. The percentage of patients with mUICC stage I, II, III, IVA, and IVB tumors was 35%, 16%, 29%, 12%, and 8%, respectively. The expression of EV‐*LINC00853* was significantly higher in patients with HCC compared to that in healthy controls, patients with CH, and patients with LC (Fig. 3A). ROC curve analysis revealed that EV‐*LINC00853* had excellent discriminatory ability [AUC = 0.934, 95% confidence interval (CI) = 0.887–0.966] in the diagnosis of HCC. The optimal cutoff value for the change in EV‐*LINC00853* expression was 14‐fold (Fig. 3B *)*. Considering the uneven age distribution between the groups, we tested whether EV‐*LINC00853* expression could vary depending on patient age. EV‐*LINC00853* expression was comparable between different age groups (Fig. S2).

**Table 1:** Baseline characteristics of patients selected for the validation cohort. Values are expressed as number (%) or mean ± SD.

Variables	Validation cohort (*N* = 182)			
	Normal (*n* = 29)	CH (*n* = 28)	LC (*n* = 35)	HCC (*n* = 90)
Age (years)	34.4 ± 7.7	46.1 ± 10.7	53.5 ± 10.5	55.1 ± 9.0
Male sex	4 (13.8)	15 (51.7)	20 (58.8)	71 (78.0)
Aspartate transaminase, IU·mL^−1^	16.62 ± 3.80	54.82 ± 52.25	82.29 ± 98.06	72.67 ± 96.80
Alanine transaminase, IU·mL^−1^	13.86 ± 7.87	67.38 ± 78.21	78.14 ± 98.10	47.92 ± 58.98
Platelet, ×10^9^/L	317 ± 35.84	185.15 ± 47.19	128.70 ± 76.64	166.41 ± 83.70
AFP, ng·mL^−1^	1.78 ± 0.68	16.65 ± 24.30	65.88 ± 132.93	4246.40 ± 14450.97
Etiology, hepatitis B virus/hepatitis C virus/alcohol/others		27 (96.4)/1 (3.6)/0/0	30 (85.7)/3 (8.6)/2 (5.7)/0	82 (91.1)/4 (4.5))/3 (3.3)/1 (1.1)
Albumin, g·L^−1^		4.55 ± 0.42	4.01 ± 0.53	4.26 ± 0.57
Bilirubin, mg·dL^−1^		0.82 ± 0.32	1.06 ± 0.99	1.39 ± 3.54
International normalized ratio		1.17 ± 0.23	1.20 ± 0.10	1.49 ± 1.89
Modified Union for International Cancer Control stage, I/II/III/IVa/IVb				32 (35)/14 (16)/26 (29) /11 (12)/ 7 (8)

### 3.4 Diagnostic performance of EV‐*LINC00853* for early‐stage HCC

**Fig. 4:**
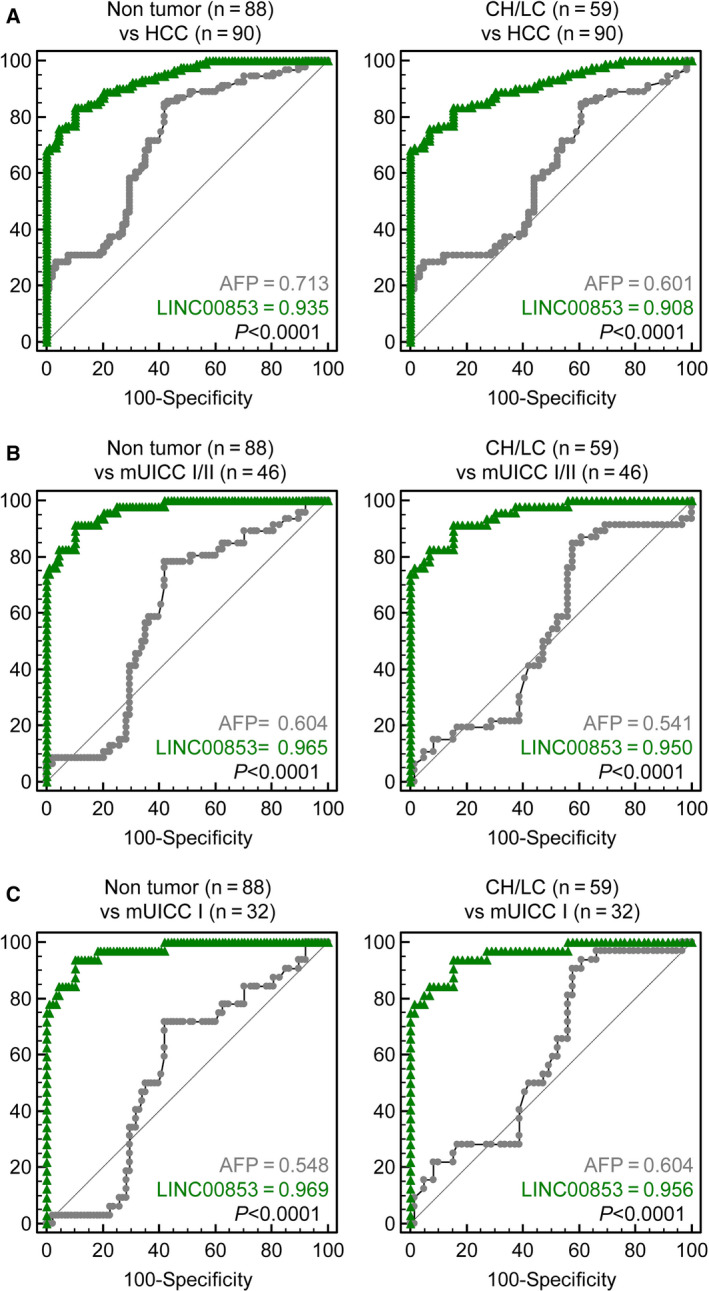
Diagnostic power of EV‐*LINC00853* in all‐stage and early‐stage HCC. (A) AUROCs for discriminating patients with all‐stage HCC from the nontumor subjects (healthy, CH, and LC) (left) and from patients at high risk of developing HCC (CH and LC) (right). (B) AUROCs for discriminating patients with mUICC stage I or II HCC from nontumor subjects (healthy, CH, and LC) (left) and from patients at high risk of developing HCC (CH and LC) (right). (C) AUROCs for discriminating patients with mUICC stage I tumors from the nontumor subjects (healthy, CHs, and LC) (left) and from patients at high risk of developing HCC (CH and LC) (right). Statistically significant differences in AUC were between EV‐*LINC00853* and AFP. Target gene expression was calculated relative to that of *HMBS*.

Next, we evaluated the diagnostic value of EV‐*LINC00853* for early‐stage HCC and compared it with the diagnostic performance of AFP. Table 2 and Fig. 4A‐C summarize the diagnostic performance and ROC curves of EV‐*LINC00853* and AFP for the diagnosis of HCC based on the tumor stage and when compared with different control groups. EV‐*LINC00853* displayed excellent discriminatory ability in the diagnosis of all‐stage HCC (Fig. 4A) as well as early‐stage HCC (Fig. 4C). The high diagnostic performance of EV‐*LINC00853* was maintained even when the control group was changed from patients without HCC to patients with CH or LC. The ROC AUC of EV‐*LINC00853* (0.908–0.969) was significantly higher than that of AFP (0.541–0.713) in all subgroup analyses (*P* < 0.001 for all comparisons). Using a 14‐fold increase as cutoff for EV‐*LINC00853* expression, and 20 ng·mL^−1^ as cutoff for the AFP level, EV‐*LINC00853* had a sensitivity of 93.75%, specificity of 89.77%, and 76.92% positive predictive value, while AFP showed only 9.38% sensitivity, 72.73% specificity, and 11.11% positive predictive value for the diagnosis of early‐stage HCC (mUICC stage I).

**Table 2:** Comparative analysis between diagnosis of HCC using serum EV‐*LINC00853* and serum AFP. PPV, positive predictive value; NPV, negative predictive value.

	*P* vs AFP	AUC	95% CI	Sensitivity (%)	Specificity (%)	PPV (%)	NPV (%)
HCC vs Nontumor							
AFP (20 ng·mL^−1^)	1	0.713	0.641–0.778	37.78	72.72	58.62	53.33
*LINC00853* (14‐fold)	< 0.0001	0.935	0.888–0.966	83.33	89.77	89.29	84.04
HCC vs CH/LC							
AFP (20 ng·mL^−1^)	1	0.601	0.517–0.680	37.78	59.32	58.62	38.46
*LINC00853* (14‐fold)	< 0.0001	0.908	0.850–0.949	83.33	84.75	89.29	76.92
mUICC I/II vs Nontumor							
AFP (20 ng·mL^−1^)	1	0.604	0.516–0.687	15.22	72.78	22.58	62.14
*LINC00853* (14‐fold)	< 0.0001	0.965	0.918–0.989	91.30	89.77	82.35	95.18
mUICC I/II vs CH/LC							
AFP (20 ng·mL^−1^)	1	0.541	0.441–0.638	15.22	59.32	22.58	47.30
*LINC00853* (14‐fold)	< 0.0001	0.950	0.889–0.983	91.30	84.75	82.35	92.59
mUICC I vs Nontumor							
AFP (20 ng·mL^−1^)	1	0.548	0.455–0.639	9.38	72.73	11.11	68.82
*LINC00853* (14‐fold)	0.1016	0.969	0.920–0.992	93.75	89.77	76.92	97.53
mUICC I vs CH/LC							
AFP (20 ng·mL^−1^)	1	0.604	0.496–0.705	9.38	59.32	11.11	54.69
*LINC00853* (14‐fold)	< 0.0001	0.956	0.891–0.988	93.75	84.75	76.92	96.15

**Fig. 5:**
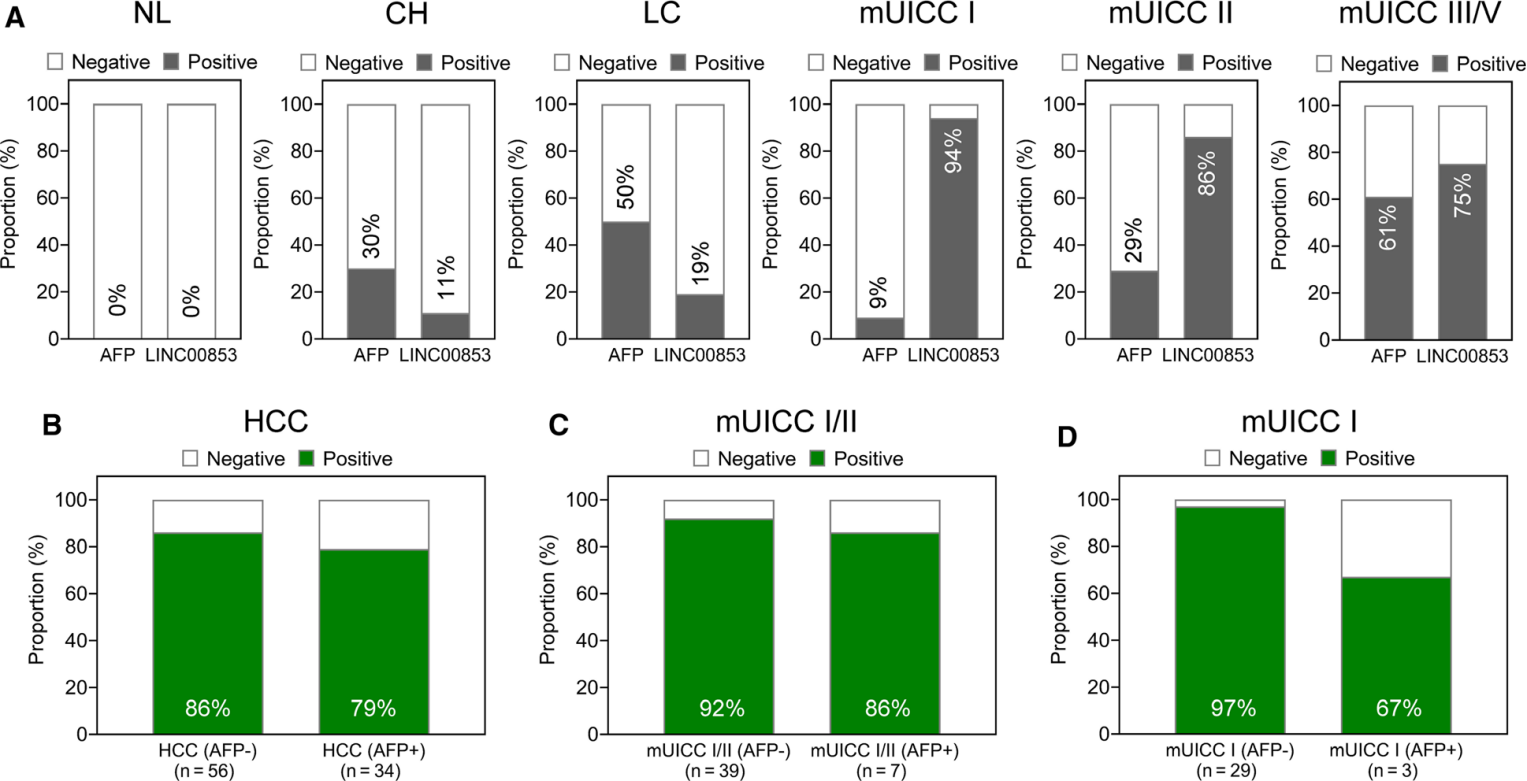
The positive rate of EV‐*LINC00853* in all‐stage and early‐stage HCC. (A) The rate of positive results of AFP and EV‐*LINC00853* in patients with each liver disease status. The cutoff for positivity was defined as a 14‐fold increase in EV‐*LINC00853* expression, and 20 ng·mL^−1^ for AFP level. (B) The rate of positive results for EV‐*LINC00853* by AFP status in patients with HCC. (C) The rate of positive results for EV‐*LINC00853* by AFP status in patients with mUICC I/II. (D) The rate of positive results for EV‐*LINC00853* by AFP status in patients with mUICC I. Target gene expression was calculated relative to that of *HMBS*.

Figure 5A compares the positivity rate of EV‐*LINC00853* and AFP in healthy subjects and in the CH, LC, and HCC groups. Interestingly, EV‐*LINC00853* had a high positivity rate even in AFP‐negative HCC (Fig. 5B–D). In mUICC stage I tumors, EV‐*LINC00853* had 97% positivity in AFP‐negative HCC and 67% positivity in AFP‐positive HCC (Fig. 5D).

### 3.5 Prognostic performance of EV‐*LINC00853* in the validation cohort

**Fig. 6:**
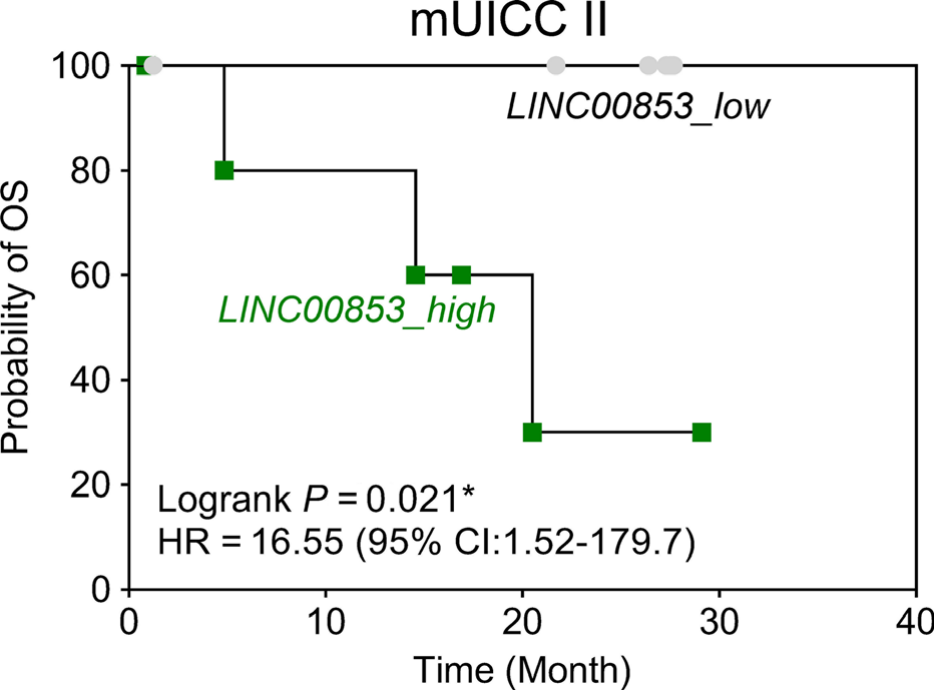
Prognostic power of EV‐*LINC00853* expression in the validation cohort. The Kaplan–Meier survival curves for overall survival based on EV‐*LINC00853* expression in patients with mUICC II HCC (Log‐rank test; *P < 0.05). Target gene expression was calculated relative to that of *HMBS*.

Previous survival analyses showed that high tissue *LINC00853* expression was associated with poor overall and disease‐free survival in TCGA_LIHC dataset (Fig. 1F). Therefore, we evaluated the prognostic power of EV‐*LINC00853* in our validation cohort. In mUICC stage II HCC, patients with high EV‐*LINC00853* expression had lower overall survival rate than those with low EV‐*LINC00853* expression (Fig. 6, HR = 16.55, 95% CI = 1.52–179.7, log‐rank *P* = 0.021). EV‐*LINC00853* expression was not associated with the overall survival rate in other‐stage HCC (Fig. S3).

## 4 Discussion

In this study, we identified seven lncRNAs that were differentially expressed between the HCC and the nontumor tissues using TCGA_LIHC data. Among them, *LINC00853* was a novel lncRNA that has never been reported in association with HCC or other malignancies. Interestingly, *LINC00853* was upregulated in serum EVs, but not in the serum of patients with HCC. In the validation cohort consisting of 90 HCC subjects and 92 non‐HCC subjects, EV‐*LINC00853* had better discriminative power in the diagnosis of all‐stage HCC and early‐stage HCC (AUC = 0.935 and 0.969, respectively) than AFP (AUC = 0.713 and 0.548, respectively).

LncRNAs exhibit tissue‐specific and species‐specific expression patterns and are also expressed in a regular manner [22], allowing them to serve as diagnostic biomarkers and prognostic factors in various disease states, including HCC [11, 23, 24, 25, 26, 27, 28, 29, 30, 31, 32, 33, 34, 35, 36, 37, 38, 39, 40, 41]. Plasma or serum *DANCR, JPX, LINC00974, LINC01225, lncRNA‐P34822, lnc‐RCDH9‐13:1, LRB1, SPRY4‐IT1, UCA1, uc003wbd, WRAP53,* and *ZFAS1* have been reported to show 51.1–92.7% sensitivity and 50.0–100% specificity for the diagnosis of HCC [26, 28, 29, 30, 31, 32, 33, 35, 36, 37, 38, 40]. Interestingly, elevated levels of *lnc‐PCDH9–13:1* can be detected in the saliva of patients with early‐stage as well as advanced‐stage HCC [40]. Accumulating evidence suggests that EV transfer of functional lncRNA between cells may play an important role in cancer development and tumor chemoresistance by altering and/or regulating local cellular microenvironments [14]. Various EV‐derived lncRNAs, including *lncRNA‐HEIH, LINC02394, LINC0635, LINC00161,* and *JPX,* are potential diagnostic biomarkers for HCC [15, 16, 17, 18].

To the best of our knowledge, our study is the first to report *LINC00853* as a novel HCC‐related EV‐derived biomarker. We believe EV‐*LINC00853* will be useful for diagnosing early‐stage tumors without elevated AFP levels. In the present study, only 9% of the early HCC (mUICC stage I) cases were AFP‐positive, while 94% of them were EV‐*LINC00853‐*positive. Moreover, 97% of the AFP‐negative early‐stage HCC cases were positive for EV‐*LINC00853*. These results contrast with those of the previous studies which reported that lncRNA expression is correlated with AFP levels [31, 38]. *LINC00853* is likely to act independently of AFP, and thus, it may be a more useful biomarker in patients with CH or LC who sometimes exhibit elevated AFP levels in the absence of HCC, leading to false‐positive results.

*LINC00853* is a 1826 nucleotide‐long lncRNA located on the chromosome 1p33. However, little is known about its biological function in disease, including malignancy. Generally, lncRNAs exert their influences via epigenetic modifications, such as chromatin modulation and DNA methylation, altering the stability of proteins and complexes, or by acting as miRNA sponge. Through these mechanisms of action, lncRNAs have been implicated in the six hallmarks of cancer: self‐sustained growth signaling, resistance to growth inhibition, avoidance of apoptosis, uncontrolled proliferation, promotion of angiogenesis, and metastasis [42]. In HCC, the lncRNA *HOTAIR* induces epigenetic silencing of the *HOXD* locus [43], *HULC* may function as a competing endogenous RNA [44], while *TERC* forms part of the catalytic center of the telomerase complex [45]. Moreover, several lncRNAs have been shown to be involved in Wnt/β‐catenin and STAT3 signaling, cancer stem cells, and epithelial‐to‐mesenchymal transition in HCC [46].

In our study, EV*‐LINC00853* expression was associated with overall survival only in patients with mUICC stage II HCC. In fact, the positivity rate of EV‐*LINC00853* decreased with increasing tumor stage, suggesting that overexpression of EV‐*LINC00853* may not reflect aggressiveness of HCC. Considering that tissue expression of *LINC00853* increased with tumor progression in TCGA_LIHC dataset, prognostic performance of EV‐LINC00853 ought to be evaluated in a larger number of patients to confirm these results.

The current study has several limitations. First, we did not investigate the functional role of EV‐*LINC00853* in HCC development. Considering that little is known about *LINC00853* and its roles in cancer, this is an area that warrants further research. Second, we did not confirm the diagnostic performance of EV‐*LINC00853* in an external patient cohort. HCC is a heterogeneous disease with various underlying etiologies, variable global prevalence, and many poorly defined prognostic patient subsets. Ours was a single‐center study whereby hepatitis B was the cause of CH, LC, and HCC in a vast majority of the subjects; thus, the results may not be directly generalizable to patient populations with different etiological backgrounds. For these reasons, large multicenter studies involving patients with a variety of liver diseases and originating from different geographical regions will be needed to comprehensively evaluate the diagnostic and the prognostic usefulness of the lncRNA biomarkers [46]. Third, we did not explore exo‐*LINC00853* expression in malignancies other than HCC; thus, we could not confirm its specificity for HCC. However, majority of the lncRNAs are highly tissue‐specific and it is possible that some may be specifically expressed in HCC, allowing for fast diagnosis and better disease management [22, 46]. Finally, due to the limited amount of human serum samples available (300 μL/sample), we were unable to use EV separation methods such as ultracentrifugation [47, 48], as recommended by MISEV2018 [49]. However, through preliminary experiments, we identified the optimal precipitation kit and RNA isolation method to use with a small amount of sample (data not shown), and confirmed that the extracted EVs satisfied the MISEV2018 criteria (Fig. 2).

## 5 Conclusions

A member of the lncRNA family, *LINC00853,* was significantly expressed in the EVs of HCC patients. EV‐*LINC00853* had excellent and significantly better discriminatory ability in the diagnosis of both all‐stage HCC and early HCC than did AFP. Furthermore, EV‐*LINC00853* showed high positivity even in AFP‐negative early HCC cases. Our findings indicate that EV‐derived *LINC00853* can serve as a potential noninvasive diagnostic biomarker for HCC that may be of particular value in patients with AFP‐negative tumors.

## Conflict of interest

The authors declare no conflict of interest.

## Author contributions

SWN, JWE, and JYC conceptualized the study. GOB and SS contributed to methodology. HRA and CWS curated the data. SSK and JWE wrote—original draft preparation. HJC wrote—review and editing. SSK, JWE, and JYC funded acquisition. All authors have read and agreed to the published version of the manuscript.

## Supporting information


**Table S1**. Seven long non‐coding RNAs overexpressed in hepatocellular carcinoma.
**Fig**.** S1**. Six known lncRNAs expression in HCC cohorts.
**Fig**.** S2**. Age‐related *LINC00853* expression in subjects without HCC.
**Fig**.** S3**. Prognostic power of EV‐*LINC00853* expression in the validation cohort.

## Data Availability

All genomic data were obtained from The Cancer Genome Atlas liver hepatocellular carcinoma project (TCGA_LIHC) and the GEO database of the NCBI (Accession Numbers: GSE94660, GSE114564, GSE124535).
