# Supplementary material for: Serum small extracellular vesicle‐derived LINC00853 as a novel diagnostic marker for early hepatocellular carcinoma
Source: Mol Oncol. 2020 Jul 13;14(10):2646–59. doi: 10.1002/1878-0261.12745 (PMC7530776; doi:10.1002/1878-0261.12745)
Supplement: Supplementary file 1 — Table S1. Seven long non‐coding RNAs overexpressed in hepatocellular carcinoma. Fig. S1. Six known lncRNAs expression in HCC cohorts. Fig. S2. Age‐related LINC00853 expression in subjects without HCC. Fig. S3. Prognostic power of EV‐LINC00853 expression in the validation cohort. [file MOL2-14-2646-s001.docx]

**Supplementary Table 1**

Seven long non-coding RNAs overexpressed in hepatocellular carcinoma

| GENE | Expression | Function | Clinical relevance in HCC | Reference |
| --- | --- | --- | --- | --- |
| SFTA1P | Up | Autophagic cell death | liver fibrosis, alcohol consumption, viral hepatitis  not associated with the HCC-specific survival | Sci Rep. 2018 Mar 29;8(1):5395 |
| HOTTIP | Up | Cell growth, metastasis | lung metastasis, tumor recurrence | Hepatology. 2014 Mar;59(3):911-23. Liver Int. 2015 May;35(5):1597-606.  PLoS Genet. 2015 Dec 28;11(12):e1005726. Hepatobiliary Surg Nutr. 2018 Dec;7(6):429-439. |
| HAGLROS | Up | cell proliferation  inhibits apoptosis,  enhances autophagy | not associated with the HCC-specific survival | Int Immunopharmacol. 2019 Aug;73:72-80.  Sci Rep. 2018 Mar 29;8(1):5395 |
| LINC01419 | Up | Unknown | HBV-related  Survival | Oncotarget. 2015 Dec 22;6(41):43770-8.  Oncotarget. 2017 Sep 28;8(56):95799-95809. |
| HAGLR | Up | Unknown | not associated with the HCC-specific survival | Sci Rep. 2018 Mar 29;8(1):5395 |
| CRNDE | Up | Proliferation, invasion  PI3K/Akt /β-catenin signaling | Survival | Am J Cancer Res. 2016 Oct 1;6(10):2299-2309.  Oncotarget. 2017 Sep 28;8(56):95799-95809.  Biomed Pharmacother. 2018 Jul;103:1187-1193. |
| LINC00853 | Unknown | Unknown | Unknown | Unknown |

**
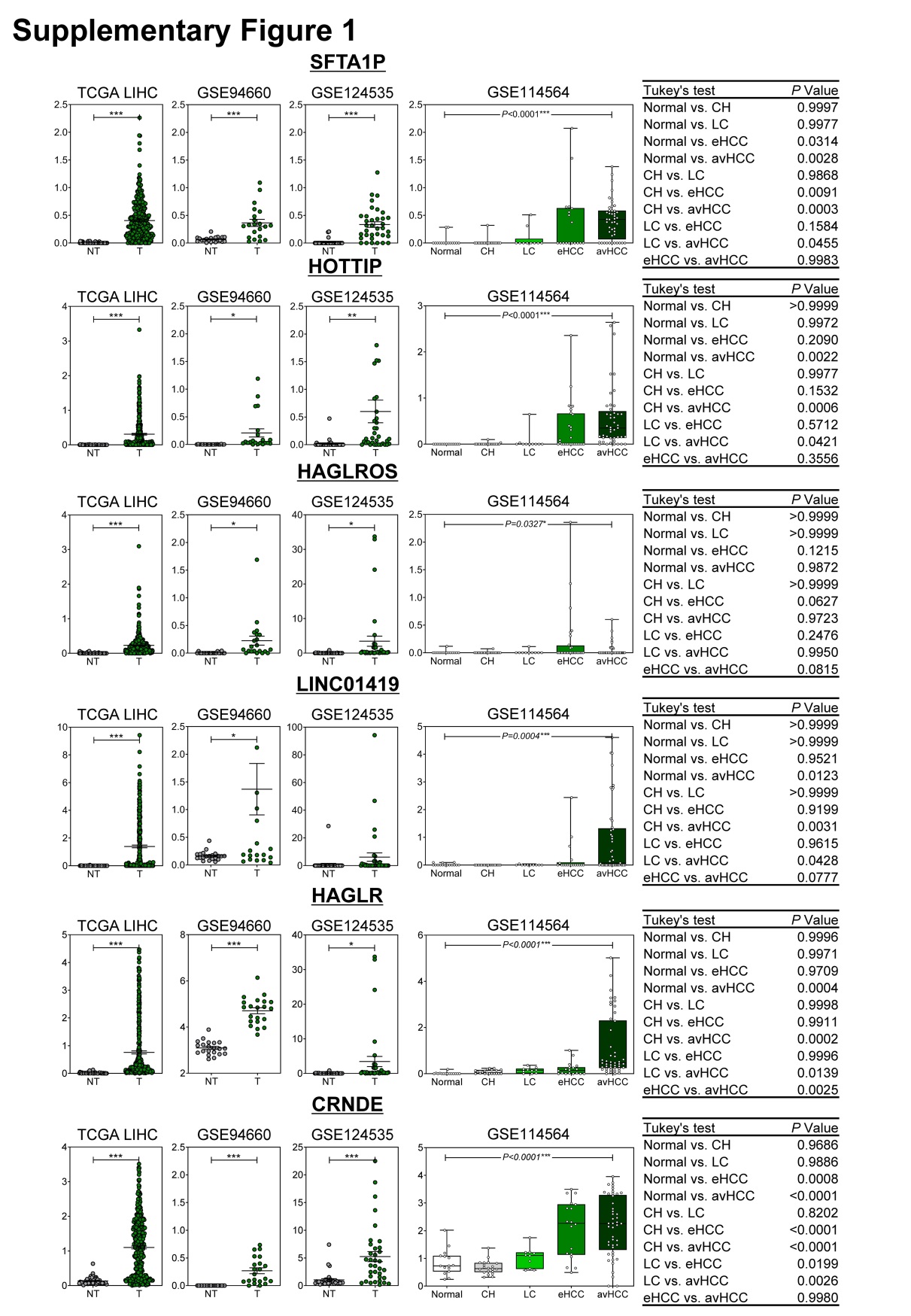
**

**Supplementary Figure 1. Six known lncRNAs expression in HCC cohorts.** Six known lncRNAs expression in the non-tumor and the HCC cohorts in four HCC RNA-Seq datasets (TCGA_LIHC, GSE94660, GSE124535, and GSE114564).


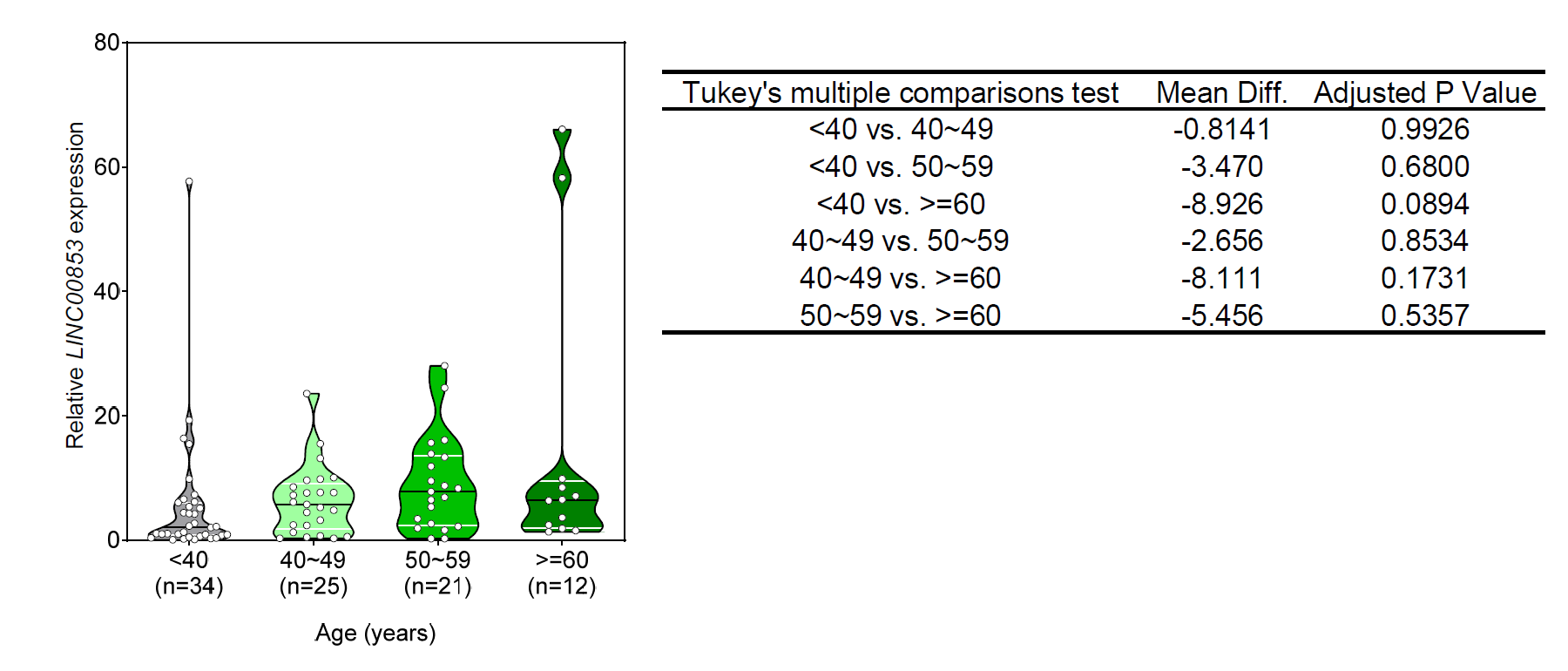


**Supplementary Figure 2. Age related *LINC00853* expression in subjects without HCC.** Target gene expression was calculated relative to that of *HMBS.*

**
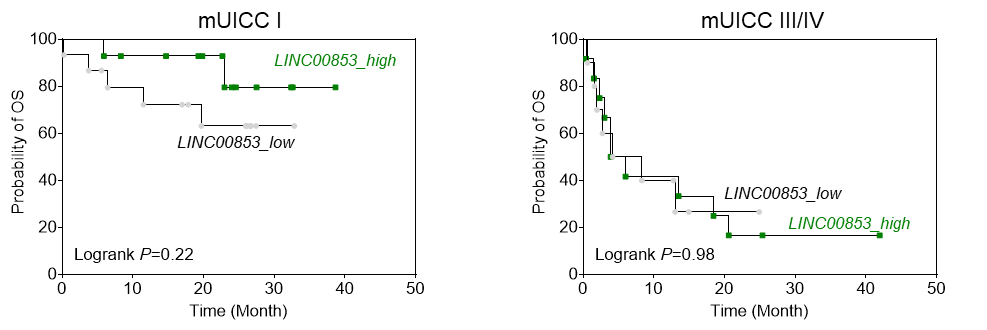
**

**Supplementary Figure 3. Prognostic power of EV-*LINC00853* expression in the validation cohort.** The Kaplan-Meier survival curves for overall survival based on EV-*LINC00853* expression in patients with mUICC I and mUICC III/IV HCC. Target gene expression was calculated relative to that of *HMBS.*
